# Nitrogen Fertilizer Rate and Crop Management Effects on Nitrate Leaching from an Agricultural Field in Central Pennsylvania

**DOI:** 10.1100/tsw.2001.91

**Published:** 2001-10-03

**Authors:** Richard H. Fox, Yuanhong Zhu, John D. Toth, John M. Jemison, Jalal D. Jabro

**Affiliations:** Department of Crop and Soil Sciences, Penn State University, University Park, PA 16802, USA

**Keywords:** nitrate leaching, nitrate pollution, modeling

## Abstract

Eighteen pan lysimeters were installed at a depth of 1.2 m in a Hagerstown silt loam soil in a corn field in central Pennsylvania in 1988. In 1995, wick lysimeters were also installed at 1.2 m depth in the same access pits. Treatments have included N fertilizer rates, use of manure, crop rotation (continuous corn, corn-soybean, alfalfa-corn), and tillage (chisel plow-disk, no-till). The leachate data were used to evaluate a number of nitrate leaching models. Some of the highlights of the 11 years of results include the following: 1) growing corn without organic N inputs at the economic optimum N rate (EON) resulted in NO_3_-N concentrations of 15 to 20 mg l in leachate; 2) use of manure or previous alfalfa crop as partial source of N also resulted in 15 to 20 mg l of NO_3_-N in leachate below corn at EON; 3) NO_3_-N concentration in leachate below alfalfa was approximately 4 mg l; 4) NO_3_-N concentration in leachate below soybeans following corn was influenced by fertilizer N rate applied to corn; 5) the mass of NO_3_-N leached below corn at the EON rate averaged 90 kg N ha (approx. 40% of fertilizer N applied at EON); 6) wick lysimeters collected approximately 100% of leachate vs. 40–50% collected by pan lysimeters. Coefficients of variation of the collected leachate volumes for both lysimeter types were similar; 7) tillage did not markedly affect nitrate leaching losses; 8) tested leaching models could accurately predict leachate volumes and could be calibrated to match nitrate leaching losses in calibration years, but only one model (SOILN) accurately predicted nitrate leaching losses in the majority of validation treatment years. Apparent problems with tested models: there was difficulty estimating sizes of organic N pools and their transformation rates, and the models either did not include a macropore flow component or did not handle macropore flow well.

## INTRODUCTION

Nitrate in leachate from agricultural fields has been a serious pollutant of the nation's waters for the last several decades. According to Swistock et al.[1], more than half of the private wells in southeastern Pennsylvania, the main agricultural area in the state, have nitrate-N concentrations above the USEPA maximum contaminant level for drinking water of 10 mg $l^{-1}$ $NO_3$-N. These authors also found that wells close to cornfields had significantly higher nitrate concentrations than those further from cornfields. The deteriorating quality of the Chesapeake Bay due to high levels of nitrate and phosphate in the bay led to the federally funded Chesapeake Bay Program that was designed to improve the bay's quality. It was determined that the main source of nitrates in the bay was agricultural fields in the watershed. More recently it was concluded that the hypoxic zone in the Gulf of Mexico at the mouth of the Mississippi is mainly due to nitrates entering the river from agricultural fields[2]. In spite of this concern with nitrate pollution from agricultural fields, few data are available showing the concentration and mass of nitrate that is leaching below agricultural fields in the Northeast and Mid-Atlantic regions of the U.S.; data on how these amounts are affected by crop rotation, N fertilizer rate, or tillage are lacking as well. Such data are also needed to calibrate and test models of nitrate leaching in agricultural fields.

An experiment was initiated in 1988 to measure the concentration and mass of nitrate leaching below manured and non-manured corn as a function of N fertilizer rate. The experiment has been continued to measure nitrate concentration and mass in leachate below alfalfa and soybeans and to determine the effect of tillage on nitrate leaching. Additional objectives were to compare the effectiveness of two lysimeter types and to determine how well several nitrate leaching models predicted the concentration and mass of nitrate in leachate.

## MATERIALS AND METHODS

The experiment was initiated in 1988 in central Pennsylvania in a silt loam soil (fine, mixed, mesic Typic Hapludalf), a well-structured soil developed from limestone parent material. There were two main treatments (manured and non-manured corn initially) with five rates of N fertilizer as the subtreatments and three replications in a randomized block design. Hand harvest determined yields of plots receiving the five N rates allowed the economic optimum N rate (EON) to be calculated using a quadratic-linear plateau model. Eighteen $0.61 \times 0.70$ m pan lysimeters were installed at a depth of 1.2 m in the low, medium, and high N fertilizer rate plots. The lysimeters were filled with polypropylene beads and forced into the roof of horizontal tunnels excavated under the crop from access pits dug into the field. Initially, there were three replications of lysimeters under the 0, 100, and 200 kg N $ha^{-1}$ fertilizer treatments of non-manured tilled corn and 0, 50, and 100 kg N $ha^{-1}$ rates of tilled manured corn[3]. Nitrate concentrations in leachate at the EON were estimated by using a linear relationship between the concentrations at the two rates bracketing the EON rate in the tilled treatments. In 1991, after 3 years of these treatments, alfalfa was planted in the manured corn block and the non-manured corn with five N rate treatments was continued[4]. In 1994, corn was planted in the herbicide-killed alfalfa treatment and the same five N rates were applied that were used for the manured corn. Beginning in 1995, the plots that had been in manured corn and alfalfa became no-till, and the plots that had been in tilled (chisel-plowed and disked) continuous corn remained the same tillage treatment. Corn was grown in 1995, 1996, 1997, and 1999 with five N rates from 0 to 200 kg N $ha^{-1}$, and soybean was grown in 1998 with no N fertilizer added[5]. Soil pH and other nutrients were maintained at optimum levels for all crops and recommended varieties and management were used to optimize yields. In 1995, eighteen $0.3 \times 0.3$ m wick lysimeters based on the design of Holder et al.[6] were installed at the 1.2 m depth in tunnels on the opposite side of the access pits[5]. The wicks were placed such that there was up to 0.5 m water tension on the lysimeter surfaces. Leachate from the lysimeters was collected weekly or after major precipitation events and the volume and nitrate concentrations were measured.

Average annual nitrate-N concentrations were calculated on a flow-weighted basis (total $NO_3^-$-N/total leachate volume). Leachate years were from May 1 to April 30 of the following year. The masses of $NO_3^-$-N lost were calculated by multiplying the $NO_3^-$-N concentration in each leachate volume collected by the pan collection efficiency corrected volume for that pan. Estimates of individual pan collection efficiencies were made using $Br^-$ tracer recovery and water balance methods[5,7].

## RESULTS AND DISCUSSION

### Nitrate Concentration in Leachate

The annual flow-weighted $NO_3^-$-N concentration in leachate below corn increased with increasing N fertilizer rate, as would be expected. The 11-year average concentration in leachate below plots receiving no fertilizer N other than that in the starter fertilizer (11 kg N $ha^{-1}$) was 4 mg $NO_3^-$-N $l^{-1}$ (Table 1). The average concentration below plots receiving the EON fertilizer rate (avg. = 182 kg $ha^{-1}$) was 17 mg $NO_3^-$-N $l^{-1}$ with a year-to-year range of 13 to 21 mg $NO_3^-$-N $l^{-1}$. The average nitrate concentration below manured corn receiving the EON was also in the 16 to 24 mg $NO_3^-$-N $l^{-1}$ range (Table 2). The $NO_3^-$-N concentration in leachate from corn following a 3-year alfalfa crop receiving the EON rate (0 broadcast N) resulted in a flow-weighted average $NO_3^-$-N concentration of 15 mg $l^{-1}$. Our results, which show that the $NO_3^-$-N concentrations in leachate from corn receiving the economic optimum N rate were always in the range of 14 to 21 mg $l^{-1}$, agree with those of similar studies using other means to estimate nitrate concentrations in leachate from optimally fertilized corn[8,9,10,11]. This relative uniformity of results appears to confirm that fertilizing corn at the economic optimum N rate will result in $NO_3^-$-N concentrations in leachate that are 3 to 11 mg $l^{-1}$ higher than the USEPA drinking water standard of 10 mg $NO_3^-$-N $l^{-1}$.

The nitrate concentrations in leachate below alfalfa averaged 4 mg $NO_3^-$-N $l^{-1}$ (Table 3), which is in good agreement with previous research showing that flow-weighted average concentrations of $NO_3^-$-N in tile drainage or leachate under alfalfa were less than 5 mg $l^{-1}$ [12,13]. The average concentration in leachate from soybean was a function of the fertilizer N rate applied to the previous corn and ranged from 5 mg $NO_3^-$-N $l^{-1}$ with 0 kg N $ha^{-1}$ of fertilizer N to 17 mg $NO_3^-$-N $l^{-1}$ with 200 kg N $ha^{-1}$ applied to the corn (Table 3). Other investigators have found that the average nitrate-N concentrations in tile drainage or leachate under soybeans in a corn-soybean rotation where corn received 136 or 224 kg N $ha^{-1}$ were 24 and 11 mg $NO_3^-$-N $l^{-1}$, respectively[12,14].

**TABLE 1**
**Average $NO_3$-N Concentration in Leachate from Non-Manured Continuous Corn at 0 and EON* Fertilizer Rates**

| Year | EON Fert. Rate (kg/ha) | Leachate $NO_3$-N Concentration (mg/L) @ O-N | EON |
|---|---|---|---|
| 1988 | 200 | 10 | 20 |
| 1989 | 175 | 9 | 21 |
| 1990 | 200 | 4 | 16 |
| 1991 | 185 | 2 | 16 |
| 1992 | 200 | 3 | 14 |
| 1993 | 177 | 2 | 14 |
| 1994 | 199 | 2 | 17 |
| 1995 | – | 5 | – |
| 1996 | 153 | 4 | 13 |
| 1997 | 187 | 2 | 17 |
| 1999 | 143 | 2 | 18 |
| **Avg.** | **182** | **4** | **17** |

* Eon = Economic Optimum N rate.

**TABLE 2**
**Average $NO_3$-N Concentrations in Leachate from Corn at the Economic Optimum N Rate for Various Managements**

| Year | EON* Fert. Rate (kg/ha) | Leachate $NO_3$-N Concentration (mg/L) at EON of Manured Corn | 1st-year Corn After Alfalfa |
|---|---|---|---|
| 1988 | 0 | 18 | |
| 1989 | 100 | 24 | |
| 1990 | 100 | 16 | |
| 1994 | 0 | | 15 |
| **Avg.** | | **19** | **15** |

* EON = Economic Optimum N Rate.

There was no statistical difference (at the 5% significance level) in 5-year average nitrate concentrations between the tilled and no-tilled treatments[5]. These results are similar to those in other published research showing that tillage had little effect on nitrate concentration in leachate or tile drainage[9,15]. There was also no statistical difference in nitrate concentrations between leachate collected with the two lysimeter types (unpublished data). However, the average leachate collection efficiency of the pan

**TABLE 3**
**Average $NO_3$-N Concentrations Leaching from Legumes**

| Year | Prev. Corn N Rate (kg/ha) | Crop | $NO_3$-N Conc. (mg/L) |
|---|---|---|---|
| 1991 | 0 | Alfalfa | 4 |
| 1992 | 0 | Alfalfa | 4 |
| 1993 | 0 | Alfalfa | 5 |
| 1998 | 0 | Soybeans | 5 |
| 1998 | 100 | Soybeans | 8 |
| 1998 | 200 | Soybeans | 17 |

lysimeters was only 40% compared to the approximately 100% collection efficiency of the wick lysimeters[5].

## Mass of Nitrate N Leached

For the 8 years where the mass of $NO_3^-$-N lost in leachate at EON could be calculated, the average amount lost was 91 kg N ha$^{-1}$ (Table 4). By subtracting the amount leached in the 0-N treatment from that leached at the EON and dividing by the economic optimum N rate of fertilizer for each year, we found that between 24 and 62% of the N fertilizer applied at the EON was lost as leachate, with an average of 40% lost. In earlier studies[16], we found that the average apparent recovery of N fertilizer by corn grain at the EON rate was 55%, so the 40% lost in leachate in the current study appears to be realistic. Other studies have reported that 41% of applied fertilizer N was lost as leachate in irrigated corn[17] and that 50% of fertilizer N applied to corn was available for leaching, denitrification, and/or $NH_3$ volatilization[18].

**TABLE 4**
**Estimated Mass of $NO_3$-N Leached from Non-Manured Corn at the Economic Optimum N Rate (EON)**

| Year | EON Rate (kg N/ha) | $NO_3$-N Leached at EON (kg N/ha) | Fert. N Leached at EON* (%) |
|---|---|---|---|
| 1988 | 200 | 108 | 29 |
| 1989 | 175 | 80 | 24 |
| 1990 | 200 | 133 | 55 |
| 1991 | 185 | 81 | 38 |
| 1994 | 199 | 55 | 25 |
| 1996 | 120 | 100 | 62 |
| 1997 | 187 | 105 | 48 |
| 1999 | 162 | 66 | 36 |
| **Avg.** | **179** | **91** | **40** |

* Fert. N leached= (N leached at EON - N leached with 0 N)/EON Rate.

## Modeling

Data from the leaching experiments were used to evaluate a number of leaching models. References to the specific models can be found in the cited papers. An overall finding was that all models could be successfully calibrated by adjusting several input parameters, but most were not that accurate in predicting nitrate leaching in validation years. Models were considered to be accurate when the mean difference between annual measured and predicted values were not significantly different from 0 at the 0.05 level. Jabro et al.[19] found that LEACHM and NCSWAP could not successfully predict nitrate leaching and that the models appeared to need a macropore-flow component and/or improved soil N submodels. We also found that LEACHMN did not predict $NO_3^-$-N leaching accurately[20] and that adding a dual-pore water-flow component to LEACHMN in LEACHMA did not markedly improve accuracy[21]. Jabro et al.[22] found that LEACHW, MACRO, NCSWAP, SLIM and SOIL could accurately predict water drainage over a 4-year period. Jabro et al.[23] also found that SOIL-SOILN accurately predicted water drainage and total annual $NO_3^-$-N leachate masses in 7 of 10 cases used in the validation set. Inaccuracy appeared to be related to inadequate modeling of N transformation processes.

## CONCLUSIONS

This research showed that the $NO_3^-$-N concentration in leachate below corn receiving the economic optimum N fertilizer rate was generally in the range of 15 to 20 mg $l^{-1}$. This agrees with other research from the northcentral and northeastern portions of the United States. However, in a typical dairy farm in Pennsylvania, where up to half of the cropland is in a forage legume such as alfalfa, which has very little nitrate leaching from it, the average nitrate concentration in leachate from the whole farm may be less than 10 mg $l^{-1}$. Corn is apparently a fairly inefficient absorber of nitrate from the soil at economic optimum N rates because approximately 40% of the N fertilizer at EON was lost as leachate. Detailed research models of nitrate leaching could be calibrated to match observed nitrate leaching results, but only one model (SOILN) accurately predicted annual nitrate-N losses in the majority (7 of 10) of validation treatment-years. Apparent problems with some models are that they have inadequate soil N transformation submodels and/or do not include macro-pore flow components.

| | | |
|---|---|---|
| Received: | June | 2, 2001 |
| Revised: | August | 27, 2001 |
| Accepted: | August | 27, 2001 |
| Published: | October | 3, 2001 |
